# Comparison of emergency surgical cricothyroidotomy and percutaneous cricothyroidotomy by experienced airway providers in an obese, in vivo porcine hemorrhage airway model

**DOI:** 10.1186/s40779-022-00418-8

**Published:** 2022-10-11

**Authors:** Tomas Karlsson, Andreas Brännström, Mikael Gellerfors, Jenny Gustavsson, Mattias Günther

**Affiliations:** 1 Department of Clinical Science and Education, Section of Anesthesiology and Intensive Care, Karolinska Institutet, 11883 Stockholm, Sweden; 2 Department of Neuroscience, Karolinska Institutet, 17177 Stockholm, Sweden; 3 Department of Physiology and Pharmacology, Section of Anesthesiology and Intensive Care, Karolinska Institutet, 11883 Stockholm, Sweden; 4 Department of Perioperative Medicine and Intensive Care, Karolinska University Hospital, 17177 Stockholm, Sweden; 5 Swedish Air Ambulance (SLA), 79291 Mora, Sweden; 6 Rapid Response Cars, 18233 Stockholm, Sweden

**Keywords:** Emergency front-of-neck airway, “Cannot intubate, cannot oxygenate” (CICO), Surgical cricothyroidotomy, Percutaneous cricothyroidotomy, Porcine model

## Abstract

**Background:**

Emergency front-of-neck airway (eFONA) is a life-saving procedure in “cannot intubate, cannot oxygenate” (CICO). The fastest and most reliable method of eFONA has not been determined. We compared two of the most advocated approaches: surgical cricothyroidotomy and percutaneous cricothyroidotomy, in an obese, in vivo porcine hemorrhage model, designed to introduce real-time physiological feedback, relevant and high provider stress. The primary aim was to determine the fastest method to secure airway. Secondary aims were arterial saturation and partial pressure of oxygen, proxy survival and influence of experience.

**Methods:**

Twelve pigs, mean weight (standard deviation, SD) (60.3 ± 4.1) kg, were anesthetized and exposed to 25–35% total blood volume hemorrhage before extubation and randomization to Seldinger technique “percutaneous cricothyroidotomy” (*n* = 6) or scalpel-bougie-tube technique “surgical cricothyroidotomy” (*n* = 6). Specialists in anesthesia and intensive care in a tertiary referral hospital performed the eFONA, simulating an actual CICO-situation.

**Results:**

In surgical cricothyroidotomy vs. percutaneous cricothyroidotomy, the median (interquartile range, IQR) times to secure airway were 109 (IQR 71–130) s and 298 (IQR 128–360) s (*P* = 0.0152), arterial blood saturation (SaO_2_) were 74.7 (IQR 46.6–84.2) % and 7.9 (IQR 4.1–15.6) % (*P* = 0.0167), pO_2_ were 7.0 (IQR 4.7–7.7) kPa and 2.0 (IQR 1.1–2.9) kPa (*P* = 0.0667), and times of cardiac arrest (proxy survival) were 137–233 s, 190 (IQR 143–229), from CICO. All six animals survived surgical cricothyroidotomy, and two of six (33%) animals survived percutaneous cricothyroidotomy. Years in anesthesia, 13.5 (IQR 7.5–21.3), did not influence time to secure airway.

**Conclusion:**

eFONA by surgical cricothyroidotomy was faster and had increased oxygenation and survival, when performed under stress by board certified anesthesiologists, and may be an indication of preferred method in situations with hemorrhage and CICO, in obese patients.

**Supplementary Information:**

The online version contains supplementary material available at 10.1186/s40779-022-00418-8.

## Background

Emergency front-of-neck airway (eFONA) is a life-saving procedure for patients who cannot be intubated or oxygenated, “cannot intubate, cannot oxygenate” (CICO), and would otherwise face imminent death. Risk factors for CICO and eFONA include obesity [1–5] and trauma [6, 7]. On the battlefield, airway compromise is the second leading cause of preventable death [8]. It is essential to identify the situation and perform eFONA before a hypoxic cardiac arrest [9]. How often CICO and eFONA occur depends on location, qualifications and experience of the health care provider and the medical condition of the patient. The incidence of eFONA varies in published literature from 0 to 18.5% for unspecified health care professionals [1, 6, 9–16]. For trauma anesthesiologists and emergency medicine doctor, the incidence is 0.2–0.3% [17, 18]. Given the infrequency of CICO, most medical personnel have little if any clinical experience of the various techniques of eFONA, and the fastest and most reliable method remains disputed. Professional societies recommend different approaches. The Difficult Airway Society (DAS) in the UK and the Swedish Society of Anesthesiology and Intensive Care recommend scalpel-bougie-tube cricothyroidotomy [1, 10, 19, 20]. The American Society of Anesthesiologists does not advocate a specific approach [21]. The Canadian Anesthesiologists’ Society, the Australian and New Zealand College of Anesthetists (ANZCA) support both needle- and scalpel-based approaches [22, 23]. The urgency and rarity of eFONA make randomized and controlled human studies challenging, which is why many studies are based on a variety of ex vivo larynx models, few simulate an obese model and results are conflicting [2, 4, 9, 24–31]. However, a real CICO situation includes a level of stress which is difficult to simulate in training [9]. Moreover, an ex vivo larynx model does not provide physiological feedback. Therefore, we introduced an obese, in vivo porcine hemorrhage model, designed to create real-time physiological feedback, relevant and high stress levels of the provider, and a proxy survival measure. We compared two of the most advocated eFONA approaches, scalpel-bougie-tube technique “surgical cricothyroidotomy” and Seldinger technique “percutaneous cricothyroidotomy” and hypothesized that amongst clinically active anesthesiologists the first would be more rapid. The primary objective was to determine the fastest method to secure airway. Secondary objectives were arterial saturation and partial pressure of oxygen, proxy survival and influence of experience.

## Methods

The study was carried out in compliance with the ARRIVE guidelines (Additional file 1). The specific pathogen free animals originated from the company Johansson, Stockholm Region, Sweden. Data and materials are available at Karolinska Institute’s electronic notebook, upon reasonable request. The animal study was approved by, and conducted in accordance with, the Swedish regional ethics approval board for animal research (approval No. 1470, reference No. 5.2.18–7487/14). The experiments were performed under veterinary supervision. Ethical approval for the human participation was not required by the Swedish Ethical Review Authority.

### Preparation

Twelve crossbred male pigs with a mean weight (standard deviation, SD) (60.3 ± 4.1) kg were anesthetized as part of a protocol of hemorrhage control, as earlier described [32, 33]. Premedication consisted of 150 mg tiletamine/zolazepam (Zoletil 100 Vet) and 6 mg medetomidine (Domitor) after which anesthesia was induced with fentanyl 2.5 µg/kg and pentobarbitalnatrium 6 mg/kg. Tracheal intubation was performed with a custom-made Miller-type laryngoscope using a standard cuffed size 8 tube. Anesthesia was maintained with ketamine 25 mg/(kg·h), midazolam 0.0485 mg/(kg·h) and fentanyl 3.5 μg/(kg·h). The animals were ventilated with a Hamilton C2 (Hamilton Medical, Geneva, Switzerland) using pressure control with initial settings positive end-expiratory pressure (PEEP) 4, peak inspiratory pressure (PIP) 15 cmH_2_O, respiratory rate 15 breaths/min and fraction of inspiratory oxygen (FiO_2_) 21%. Settings were adjusted to achieve normoventilation (pCO_2_ 4.9–5.7 kPa). Arterial blood gases [pH, pCO_2_, pO_2_, lactate, arterial blood saturation (SaO_2_) and base excess (BE)] were collected at baseline and at secure airway (GEM Premier 4000, Instrumentation Laboratories, Lexington, MA, USA). The animals were exposed to 25–35% of total blood volume hemorrhage and Resuscitative Endovascular Balloon Occlusion of the Aorta or aortic tourniquet intervention. Hemorrhage was achieved through surgical exposure and then transection of the common femoral artery or the carotid artery. It took approximately 20 min to reach the estimated blood loss in both groups [32, 33]. At completion of the primary studies, the animals were stabilized for 30 min in the ventilator. The inclusion criteria which had to be fulfilled were sinus-rhythm, SaO_2_ > 95% by FiO_2_ 0.21, pCO_2_ 4.5–6 kPa and systolic arterial pressure (SAP) > 80 mmHg. All animals that were assessed for eligibility met the inclusion criteria. Animals were given the neuromuscular blocking drug rocuronium bromide (0.6 mg/kg) to prevent from spontaneous ventilation which could confound the results. The trial included a trial leader/procedure assistant, a cameraman, an assistant for data recording and an assistant for blood sampling and the doctor randomized to perform the procedure.

### Experimental setup

Twelve specialists in anesthesiology and intensive care in a tertiary referral hospital were undertaking health care service during the day and taken from regular service without prior notification. Oral consent was obtained immediately before the trial for participation and filming. To ensure a baseline level of training in these techniques, all anesthesiologists were required to be board certified specialists in anesthesia and intensive care and pass a structured one-day airway management team training course not later than 4 years before the study. The course included lectures, dry lab benchtop instructions, practice of the two eFONA techniques in the study and dry lab simulation scenarios with video analyses. Mean provider experience, defined as total years of full-time anesthesia service including residency was recorded. The previous eFONA experience of the participating anesthesiologists is displayed in Additional file 2: Table S1. The anesthesiologists received instructions about the experimental CICO situation and were given a brief oral refresher about the eFONA methods by the trial leader. The trial was double-blinded until randomization, which was done by one permuted-block and allocation by random numbers to either Seldinger technique (percutaneous cricothyroidotomy, *n* = 6) or a scalpel-bougie-tube technique (surgical cricothyroidotomy, *n* = 6). Tags were hidden in an opaque sealed container. A CICO was declared, the animal extubated and the anesthesiologist permitted to approach and palpate the animal and start the procedure. Timing began at the start of palpation and stopped when a secure airway was established (auscultative thoracic respiratory sounds). The tracheal tube was then connected to the ventilator for verification of an end-tidal capnogram. A predefined time-limit was set to 360 s (6 min). The reasons were the following: (1) Loss of aortic fluctuation occurs after 3–4 min [34, 35] in asphyxia cardiac arrest in swine and rats. With an additional 2 min, we deemed this time span appropriate for determining circulatory collapse due to asphyxia. It is an approximation, as it is not possible to determine an exact time when a cardiac arrest is irretrievable. (2) A shorter time span could cause a reversible cardiac arrest, and a longer time span would be less clinically relevant and not in accordance with the ethical permit for animal euthanasia. At completion, the animals were euthanized with 60 ml pentobarbitalnatrium (Alfatal Vet 100 mg/ml) and postmortem examinations were performed. The length of the skin incision and the depth from the skin to the cricothyroid membrane were measured. Macroscopically detectable complications were documented. All procedures were filmed for verification of timings and procedural sequences. Time of onset of cardiac arrest was defined as start of pulseless electrical activity (PEA). PEA was defined as a blood pressure trace below 5 mmHg from peak to bottom and was calculated retrospectively. Cardiac output is insufficient to maintain adequate organ perfusion at this pressure [36]. Animals with onset of cardiac arrest were defined as non-survivals.

### eFONA approaches

**Fig. 1:**
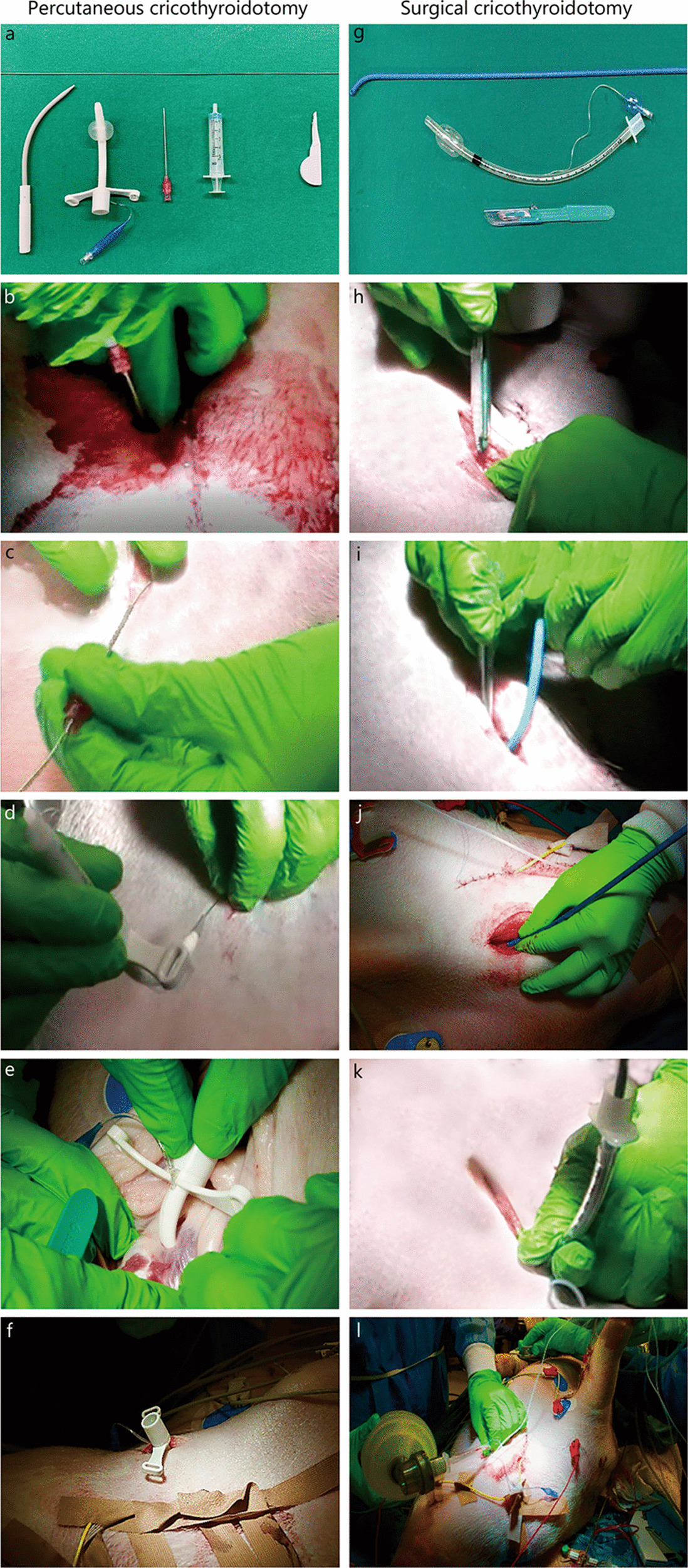
Overview of the equipment and time sequence of the two techniques percutaneous cricothyroidotomy and surgical cricothyroidotomy. The percutaneous cricothyroidotomy equipment is used for (**a**) cannulation of cricothyroid membrane, after vertical incision by scalpel (**b**) followed by guidewire through cricothyroid membrane and subsequent cannula removal from guidewire (**c**), onto which the Melker cricothyrotomy tube with curved dilator is slid (**d**) to end with the cricothyrotomy tube in the final position, after dilator and guidewire have been removed (**e**). The cricothyrotomy tube through cricothyroid membrane (**f**). The surgical cricothyroidotomy equipment is used for (**g**) vertical incision by scalpel (**h**) followed by insertion of Frova introducer perpendicularly to cricothyroid membrane whilst scalpel maintaining open the membrane (**i**), after which the introducer is slid down in trachea (**j**), with subsequent tracheal tube railroaded over introducer into trachea (**k**). Tracheal tube in final position, ventilation with bag-valve (**l**)

The percutaneous cricothyroidotomy consisted of a Melker Cricothyrotomy set (Cook Medicals, Bloomington, IL, USA, Fig. 1a). It was performed by an initial incision in the skin followed by palpation and needle perforation of the cricothyroid membrane, with simultaneous aspiration in a 5 ml syringe. After verification of air in the syringe, it was detached from the cannula. A guidewire was inserted through the cannula into the airway. The cannula was thereafter removed. The assembled tracheostomy tube and dilator were inserted over the guidewire and dilated through the cricothyroid membrane. The dilator and guidewire were then removed as one unit from the tube and the position was verified by ventilation with a bag-valve connected to the tube (Fig. 1b–f).

The surgical cricothyroidotomy consisted of a size “20 safety scalpel (BD Bard-Parker)”, a Frova Intubating Introducer (Cook Medicals, Bloomington, IL, USA) and a standard Rüsch cuffed tracheal tube size 6 (Teleflex, Morrisville, NC, USA, Fig. 1g). The 20 safety scalpel was chosen because, in our experience, this improved the incision through the pigskin and the introduction of the bougie into the trachea. Palpation and a “laryngeal handshake” were followed by a vertical incision in the skin and subcutaneous tissue. Blunt dissection was performed to reach the cricothyroid membrane which was transversally stab incised. A 90 degree rotation with the sharp edge caudally was performed. While keeping the blade inside the airway with the non-dominant hand, a Frova introducer was inserted, with the tip perpendicular to the airway. The introducer was rotated in a fan shape while pushing the catheter distally. When in place in trachea, the tube was railroaded over the introducer and advanced down until the cuff disappeared. The cuff was inflated, and the animal was ventilated with a bag-valve connected to the tube (Fig. 1 h–l).

Both approaches were considered optimal for these techniques, and only minor corrections were needed during the experiments.

### Statistical analyses

Statistical analyses were performed using GraphPad Prism version 9.0 (GraphPad Software, La Jolla, CA). Normality of distribution was assessed using the Shapiro–Wilk test. All data were normally distributed. Because a cut-off was set to 360 s in the primary endpoint, time to secure airway, the data were analyzed by the non-parametric Mann–Whitney. To make data presentation and analysis consistent, and to account for the small sample size, all data except for the repeated measures was presented as median and IQR. SaO_2_, pO_2_, pCO_2_, pH, BE and lactate were analyzed with multiple Mann–Whitney tests with False Discovery Rate adjusted *P*-value (Benjamini, Krieger and Yekutieli). SAP and heart rate were repeated measures, and therefore analyzed with mixed effects analysis and presented with mean ± SD. Provider experience was analyzed by Mann–Whitney. Time to secure airway vs. years in anesthesia was done by simple linear regression. *P* < 0.05 was considered statistically significant.

## Results

**Fig. 2:**
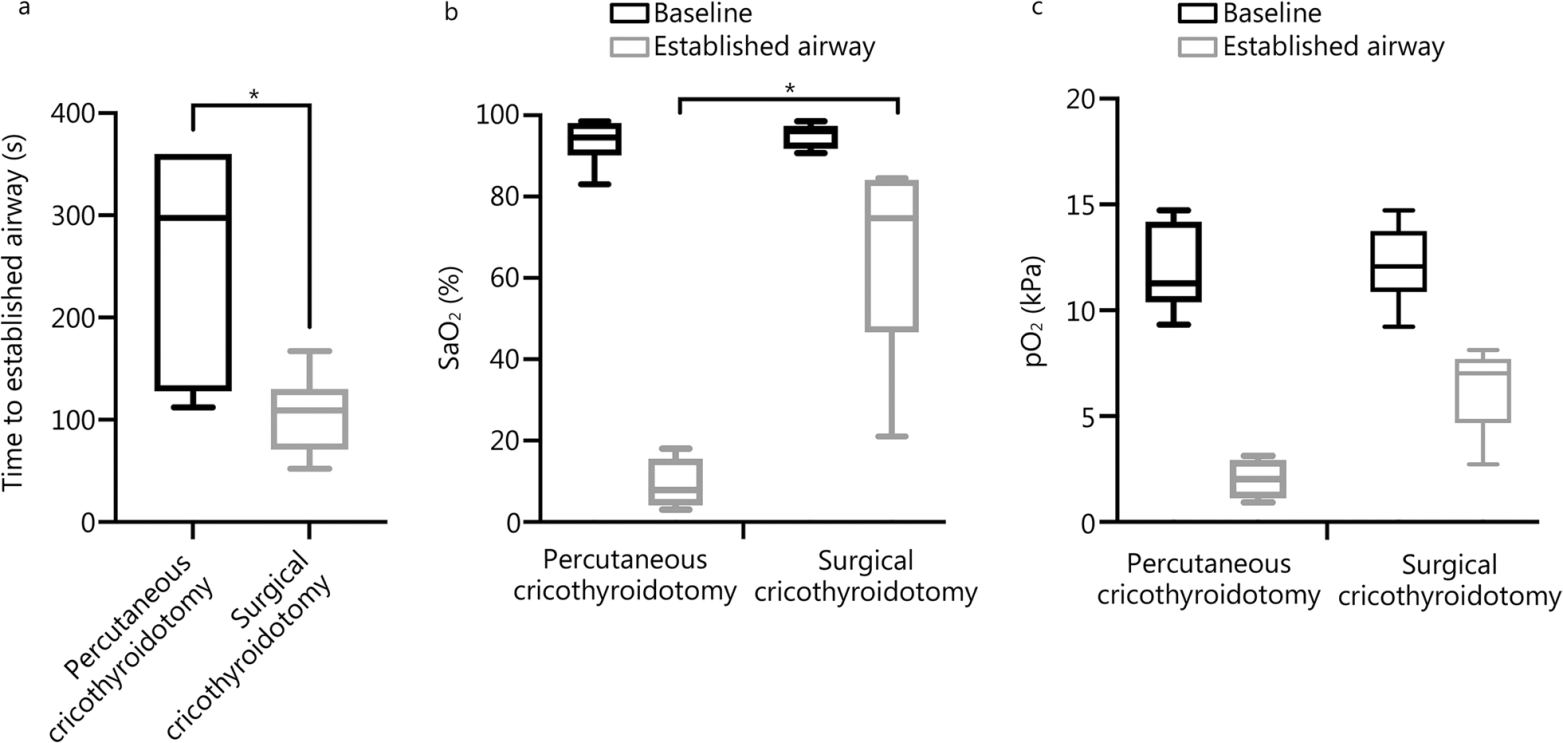
Multipanel graphs showing the difference in time to secure airway (**a)**, SaO_2_ (**b**) and pO_2_ (**c**), between the groups. Time to established airway decreased and saturation at established airway increased after surgical cricothyroidotomy. ^*^P < 0.05

**Fig. 3:**
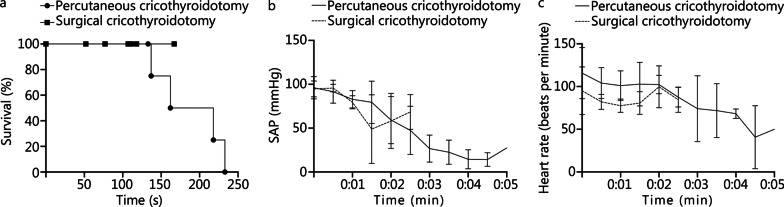
Multipanel graphs showing Kaplan-Meyer survival (**a**), systolic arterial pressure (SAP) (**b**) and heart rate (**c**) in the two groups. As SAP decreased, the survival rate diminished in the percutaneous cricothyroidotomy group. No difference was detected in heart rate

**Fig. 4:**
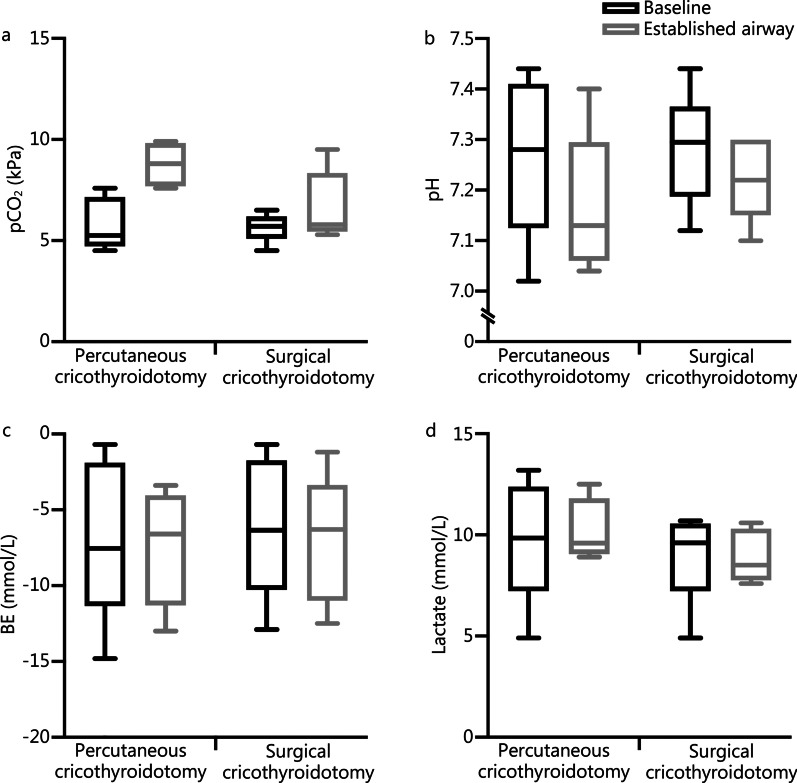
Multipanel graphs showing the difference in pCO_2_ (**a**), pH (**b**), base excess (BE) (**c**) and lactate (**d**), between the groups. No differences were detected in the established airway in comparison of the groups

At baseline, no differences were detected between groups in SAP, heart rate, SaO_2_, pO_2_, pCO_2_, pH, BE or lactate. Median (IQR) time to secure airway was 109 (IQR 71–130) s in surgical cricothyroidotomy and 298 (IQR 128–360) s in percutaneous cricothyroidotomy (*P* = 0.0152, Fig. 2a). At established airway, SaO_2_ was lower in percutaneous cricothyroidotomy, 7.9 (IQR 4.1–15.6) %, compared with surgical cricothyroidotomy, 74.7 (IQR 46.6–84.2) % (*P* = 0.0167, Fig. 2b). pO_2_ was lower, although it did not reach statistical significance, in percutaneous cricothyroidotomy, 2.0 (IQR 1.1–2.9) kPa, compared with surgical cricothyroidotomy, 7.0 (IQR 4.7–7.7) kPa (*P* = 0.0667, Fig. 2c). In percutaneous cricothyroidotomy, two anesthesiologists failed to secure the airway and the time was set to 360 s, and data analyzed by non-parametric methods. All animals survived surgical cricothyroidotomy. Four animals did not survive percutaneous cricothyroidotomy. Times of cardiac arrest were 137–233 s, 190 (IQR 143–229), from declared CICO (Fig. 3a). No differences were detected between groups in SAP or heart rate (Fig. 3b, c). There were no differences in pCO_2_, pH, BE or lactate in established airway (Fig. 4).

**Fig. 5:**
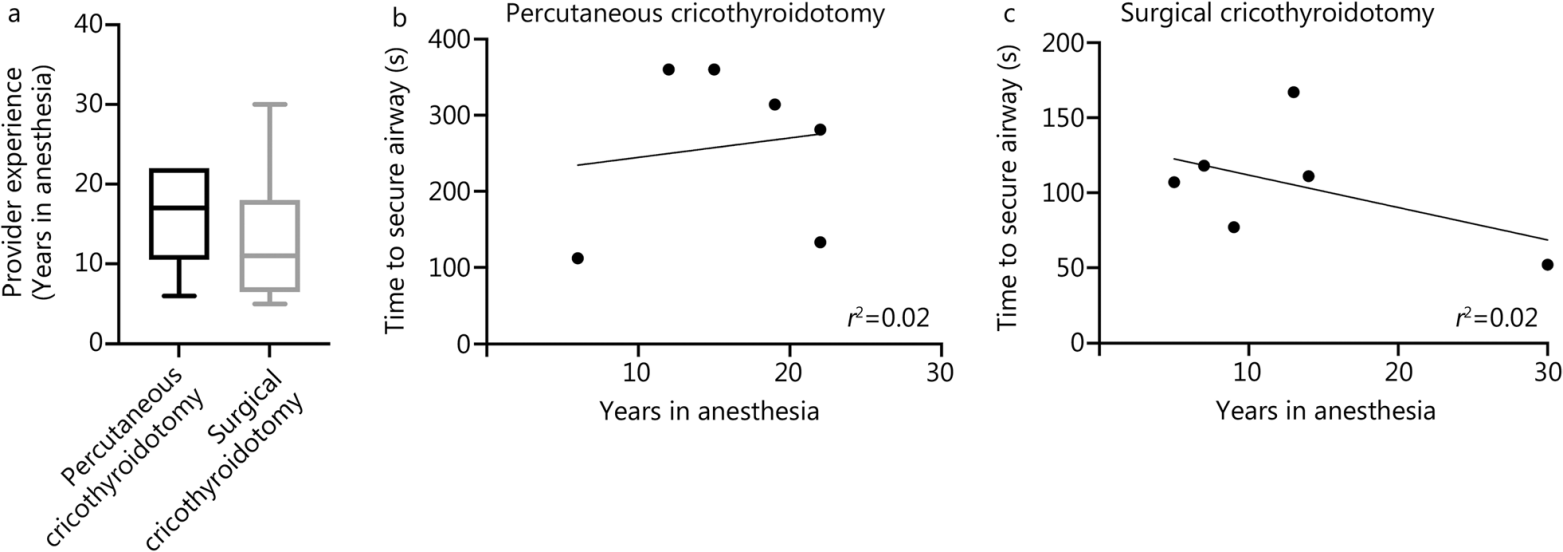
Multipanel graphs showing the difference in provider experience between the groups (**a**), linear regression of time to secure airway in relation to years in anesthesia for the Seldinger technique (**b**) and linear regression of time to secure airway in relation to years in anesthesia for the surgical technique (**c**). No differences were detected between groups

Median (IQR) years in anesthesia were 13.5 (IQR 7.5–21.3). Median (IQR) provider experience was for percutaneous cricothyroidotomy 17.0 (IQR 10.5–22.0) years and surgical cricothyroidotomy 11.0 (IQR 6.5–18.0) years. There was no detectable difference in their time in anesthesia between groups (*P* = 0.37, Fig. 5a). Time to secure airway was not correlated to their years in anesthesia, in the percutaneous cricothyroidotomy (*P* = 0.78) or surgical cricothyroidotomy (*P* = 0.32, Fig. 5b, c). Additional file 2: Table S1 describes their previous experience of eFONA and surgical airways. Among the doctors, 17% (2/12) had performed eFONA. The percutaneous tracheostomy experience varied, with one group of doctors having performed 1 to 5 procedures and one group with more than 20 procedures. The most experienced group consisted of 4 individuals. One-third had previously performed surgical tracheostomies. Mean ± SD depth from the skin to the cricothyroid membrane was (3.0 ± 0.5) cm. In surgical cricothyroidotomy, all skin incisions were vertical with (7.5 ± 2.8) cm. In percutaneous cricothyroidotomy, 5 skin incisions were vertical with (5.2 ± 1.3) cm and one made two skin incisions that were horizontal with 2.5 cm and 4 cm respectively. Adverse events for surgical cricothyroidotomy were one moderate bleeding from a complete lesion of thyroid gland, two minor bleedings from the tracheal tube with unknown source, two lesions of the extrinsic laryngeal muscles, one lesion of posterolateral tracheal wall and one incision between the cricoid cartilage and the first tracheal ring. Adverse events for percutaneous cricothyroidotomy were one major bleeding from unknown source, one insertion of the tracheostomy through the laryngeal cartilage, one double needle and/or guidewire perforation and one lesion of the posterior tracheal wall, one tracheostomy inserted posteriorly to the trachea and five multiple needle punctures.

## Discussion

In this animal study, eFONA by surgical cricothyroidotomy was faster and had improved proxy survival and oxygenation compared to percutaneous cricothyroidotomy. The study was designed to follow hemorrhage control studies according to our previous works [32, 33], which was ethically beneficial by honoring the 3R of animal welfare in research by “reducing the number of animals used to a minimum and to obtain information from fewer animals or more information from the same number of animals”. We considered the model relevant to simulate a CICO situation. Hemorrhage occurs in trauma, and trauma patients and obese patients have higher prevalence of CICO [2, 17]. However, the design also introduced possible confounders by the previous experimental procedures. Therefore, animals were included only if fulfilling a set of predefined physiological criteria, and the airway randomization was performed independently of the preceding studies.

Survival was 100% in the surgical cricothyroidotomy group and 33% in the percutaneous cricothyroidotomy group. By our definition, onset of cardiac arrest occurred between 137 and 233 s. Survival in this context should be considered as an indication of clinical outcome, in addition to the primary outcome: timing of the procedure, as we did not determine the reversibility of the PEA. The heart rates did not decrease below 50 beats per minute in any group. This contradicts the experience in humans [37], and the heart rate reaction may be specific to the porcine model, or interference from the ketamine-anesthesia.

The in vivo design introduced clinically relevant stress levels in the providers, simulating a true CICO situation. The stress was induced by an unpreparedness for the CICO combined with continuous clinical feedback of desaturation and hypotension during the procedure. Therefore, we did not perform a cross-over design, in comparison to studies without stress induction [2, 24, 28, 30, 38, 39]. We intended to test a comparable group of clinically active anesthesiologists, as anesthesiologists may often be the primary responders to unexpected CICO situations. Earlier reports have included various training levels [4, 9, 39]. Years of experience of the anesthesiologists did not differ between groups, and time to secure airway was not correlated to years of providers in anesthesia in either group. Interestingly, most anesthesiologists experienced difficulties in performing percutaneous cricothyroidotomy, irrespective of clinical experience, and the least experienced participant performed percutaneous cricothyroidotomy the most appropriately.

SaO_2_ and pO_2_ were lower, although pO_2_ did not reach statistical significance, after percutaneous cricothyroidotomy compared to surgical cricothyroidotomy. pCO_2_ remained unaffected, which suggests that hypoxia and not hypercapnia may be time limiting in CICO. pH, BE and lactate were unaffected, which corresponds to respiratory acidosis not being a limiting factor in CICO [40].

The mean length of incision was 5.2 cm in percutaneous cricothyroidotomy, and the initial incision had to be extended in most attempts, reflecting the difficulty of the procedure. The mean length of incision in surgical cricothyroidotomy was 7.5 cm. The DAS guidelines recommend an 8 to 10 cm incision in obese patients [19]. Anatomical differences between humans and pigs also include a thicker frontal neck section, no chin protrusion, and a longer distance from head to the thorax. The thyroid gland is located more distally, potentially reducing bleeding complications. The larynx anatomy is generally larger, while the cricothyroid interval is deeper [41, 42]. It was not possible to palpate any structures other than the existence of the laryngeal cartilage. The mean depth from the skin to the cricothyroid membrane was 30 mm, while 18 to 45 mm is considered to be obese in humans. Therefore, pigs of this size may be described as obese neck models [2, 4, 43, 44]. Obese neck models are relevant in advanced airway management, as the risk of CICO increases in patients with difficult neck anatomy [2, 30, 31, 39]. The increased number of adverse events in percutaneous cricothyroidotomy was likely related to the difficulty of accessing the trachea. Multiple attempts were needed which led to increased stress, time consumption and subsequent failures. In comparison, only one serious complication was detected in surgical cricothyroidotomy: hemorrhage from a lesioned thyroid gland. Our findings contrast earlier findings of high failure and complication rates of surgical cricothyroidotomy [39].

To our knowledge, this is the first study introducing an in vivo, obese neck model, with real-time physiological feedback. The time to secure airway was 109 s, similar to Le Fevre’s obese manikin models [2] and faster than 171.5 s in Howes morbidly obese manikins with standard surgical technique [4]. The results contrast the difficult ovine airway model by Heard, which had 86 s for a cannula based eFONA and a 100% success rate [38]. Heard et al. [38] injected one liter of crystalloid fluid into the sheep’s anterior neck, which may result in looser tissue in comparison to the dense subcutaneous tissue in our model. Also, the reuse of animals by Heard et al. [38] may have affected the performance of the participants. Time to secure airway in obese studies thus far, have all demonstrated a time consumption that could lead to irreversible organ damage. Faster eFONA may require new methods such as the “Cric guide” [45] or the use of ultrasound which enhance accuracy in obese patients [46]. Frequent practice using standard techniques in live, obese neck models with real-time physiological feedback may also improve confidence, accuracy and speed. We suggest that future research may include obese models and ultrasound.

The study has some limitations. First, although the anesthesiologists had equal baseline training in the hospital and all doctors came from the same department of anesthesiology and intensive care, the previous experience of FONA and surgical airways differed. Two doctors had clinical experience of eFONA, which occurred several years before the trial and the two most recent procedures involved the Melker kit. These doctors were randomized to the Seldinger group. Five doctors had more experience of percutaneous tracheostomy and surgical tracheostomies. Four of these were randomized to the Seldinger group. Despite the randomization of doctors with experience of Seldinger to the Seldinger group, the results were in favour of surgical eFONA. This lowered the risk for a type two error, failure to detect a difference between groups. In addition, a course in eFONA for the anesthesiologists before the study would have interfered with the experimental intention of an unprepared eFONA. Therefore, while the study indicates the fastest method in an actual CICO, the methods should be further investigated in prospective, randomized trials. Second, there was a potential risk of selection bias in favor of the percutaneous technique due to the relatively greater familiarity of Seldinger than surgical approaches amongst anesthesiologists. Third, the trial was not a cross-over design. The reason was that the risk for selection bias would be unacceptable in a cross-over design, in this specific experimental setup as discussed above. Fourth, while the swine may represent an obese neck model, there are inherent differences in anatomy between the swine and humans, specifically the length of the front-of-neck, the lack of a protruding chin and the thyroid gland location, as discussed above. Fifth, we used a size 20 scalpel which we believe optimized the methodology in the pig, still it’s a limitation as size 10 is standard in humans. Sixth, there are many percutaneous techniques described [32, 33]. This study may therefore not draw conclusions of the performance of other techniques, although the difficulties of cricothyroid membrane perforation suggest that other percutaneous techniques would perform similarly. Seventh, the sample size was small, which is why the correlation between experience and performance of the methods should be corroborated in future larger trials.

## Conclusions

eFONA by surgical cricothyroidotomy using the scalpel-bougie-tube technique was faster and had increased oxygenation and survival compared to percutaneous cricothyroidotomy using the Seldinger technique, when performed under high stress by board certified anesthesiologists. Years in anesthesia did not correlate to success in any technique. The percutaneous cricothyroidotomy had increased risk of failure, time consumption and adverse events. eFONA by surgical cricothyroidotomy may be an indication of preferred method in situations with hemorrhage and CICO in obese patients.

## Supplementary Information

**Additional file 1**: The ARRIVE guidelines.

**Additional file 2**: **Table S1**. eFONA and surgical airway experience of participating anesthesiologists.

## Data Availability

Not applicable.

## Abbreviations

ANZCA: Australian and New Zealand College of Anesthetists
BE: Base excess
CICO: Cannot intubate, cannot oxygenate
DAS: Difficult Airway Society
eFONA: Emergency front-of-neck airway
FiO_2_: Fraction of inspiratory oxygen
PEA: Pulseless electrical activity
PEEP: Positive end-expiratory pressure
PIP: Peak inspiratory pressure
SaO_2_: Arterial blood saturation
SAP: Systolic arterial pressure
