# Supplementary material for: Comparison of emergency surgical cricothyroidotomy and percutaneous cricothyroidotomy by experienced airway providers in an obese, in vivo porcine hemorrhage airway model
Source: Mil Med Res. 2022 Oct 11;9:57. doi: 10.1186/s40779-022-00418-8 (PMC9552401; doi:10.1186/s40779-022-00418-8)
Supplement: Supplementary file 2 — Additional file 2: Table S1. eFONA and surgical airway experience of participating anesthesiologists. [file 40779_2022_418_MOESM2_ESM.pdf]

Table S1 eFONA and surgical airway experience of participating anesthesiologists

Doctor	eFONA (*n*), years since performed (y)	Percutaneous tracheostomy (*n*)	Surgical tracheostomy (*n*)	Percutaneous allocation	Surgical allocation
1	0	4	0		X
2	2, 6	20 - 30	0	X	
3	10, >10	> 30	3	X	
4	0	1	0	X	
5	0	2	0		X
6	0	3	0		X
7	0	5	0	X	
8	0	2	0		X
9	0	3	0		X
10	0	5	5	X	
11	0	20 - 30	5		X
12	0	20 - 30	10	X	

X indicates randomized group allocation of doctor. *eFONA* emergency front-of-neck airway
